# Hypothyroidism and risks of cerebrovascular complications among patients with head and neck cancer after radiotherapy

**DOI:** 10.1186/s12883-021-02047-5

**Published:** 2021-01-19

**Authors:** Chi-Hung Liu, Joseph Tung-Chieh Chang, Tsong-Hai Lee, Pi-Yueh Chang, Chien-Hung Chang, Hsiu-Chuan Wu, Ting-Yu Chang, Kuo-Lun Huang, Chien-Yu Lin, Kang-Hsing Fan, Yeu-Jhy Chang

**Affiliations:** 1 Stroke Center and Department of Neurology, Chang Gung Memorial Hospital, Linkou Medical Center and College of Medicine, Chang Gung University, Taoyuan, Taiwan; 2 Department of Radiation Oncology, Chang Gung Medical Foundation, Linkou Chang Gung Memorial Hospital, Proton and Radiation Therapy Center, Taoyuan, Taiwan, and Department of Medicine, College of Medicine, Chang Gung University, Taoyuan, Taiwan; 3 Taipei Chang Gung Head & Neck Oncology Group, Chang Gung Memorial Hospital Linkou Medical Center, Taoyuan, Taiwan; 4 Department of Laboratory Medicine, Chang Gung Memorial Hospital, Linkou Medical Center, Taoyuan, Taiwan; 5 Department of Medical Biotechnology and Laboratory Science, Chang Gung University, Taoyuan, Taiwan; 6 Particle Physics and Beam Delivery Core Laboratory of Institute for Radiological Research, Chang Gung University / Chang Gung Memorial Hospital, Linkou, Taoyuan, Taiwan; 7 Chang Gung Medical Education Research Centre, Taoyuan, Taiwan

**Keywords:** Carotid artery stenosis, Head and neck cancer, Hypothyroidism, Ischemic stroke, Radiation therapy

## Abstract

**Background:**

Hypothyroidism (HT) and carotid artery stenosis (CAS) are complications of radiotherapy (RT) in patients with head and neck cancer (HNC). The impact of post-RT HT on CAS progression remains unclear.

**Methods:**

Between 2013 and 2014, HNC patients who had ever received RT and were under regular follow-up in our hospital were initially screened. Patients were categorized into euthyroid (EU) and HT groups. Details of RT and HNC were recorded. Total plaque scores and degrees of CAS were measured during annual extracranial duplex follow-up. Patients were monitored for CAS progression to > 50 % stenosis or ischemic stroke (IS). Cumulative time to CAS progression and IS between the 2 groups were compared. Data were further analyzed based on the use or nonuse of thyroxine of the HT group.

**Results:**

333 HNC patients with RT history were screened. Finally, 216 patients were recruited (94 and 122 patients in the EU and HT groups). Patients of the HT group received higher mean RT doses (HT vs. EU; 7021.55 ± 401.67 vs. 6869.69 ± 425.32 centi-grays, *p* = 0.02). Multivariate Cox models showed comparable CAS progression (*p* = 0.24) and IS occurrence (*p* = 0.51) between the 2 groups. Moreover, no significant difference was observed in time to CAS progression (*p* = 0.49) or IS (*p* = 0.31) among patients with EU and HT using and not using thyroxine supplement.

**Conclusions:**

Our results did not demonstrate significant effects of HT and thyroxine supplementation on CAS progression and IS incidence in patients with HNC after RT.

## Background

Radiation-induced thyroid disorders are well-known complications after radiotherapy (RT) in patients with head and neck cancer (HNC) [1, 2]. The thyroid gland is a major endocrine organ producing thyroid hormones for maintaining metabolism. Injury to the thyroid gland due to radiation may induce short-term thyroiditis and hypothyroidism (HT). Moreover, post-RT HT is associated with the accumulative radiation dose to the thyroid gland [3]. The cumulative incidence of post-RT HT, including clinical and subclinical HT, may increase to 50 % with time following RT [1, 4].

HT may increase atherogenic risks through increased blood pressure and low-density lipoprotein cholesterol, homocysteine, and high-sensitivity C reactive protein levels [5]. Studies have shown that even subclinical HT may be associated with greater carotid intima-media thickness [6–8]. Although stroke risks may not increase grossly in patients with subclinical HT, they could be higher in young patients with subclinical HT [9]. Studies have demonstrated that thyroxine supplement may slow down the progression of carotid intima-media thickness [8, 10]. Furthermore, in HNC patients, carotid artery stenosis (CAS) following RT is a common complication [11, 12]. Moreover, the incidence of radiation vasculopathy increases over time and is associated with accumulative radiation doses [13]. Similarly, stroke risks may increase after RT [14]. Therefore, regular carotid artery surveillance should be considered in HNC patients after RT. However, few researchers have discussed the association between post-RT HT and post-RT vasculopathy. Whether treating HT could alleviate CAS progression is unclear. Herein, we discuss the associations between post-RT HT, stroke, and CAS progression in our HNC patients.

## Methods

### Patient and demographic data recruitment

**Fig. 1:**
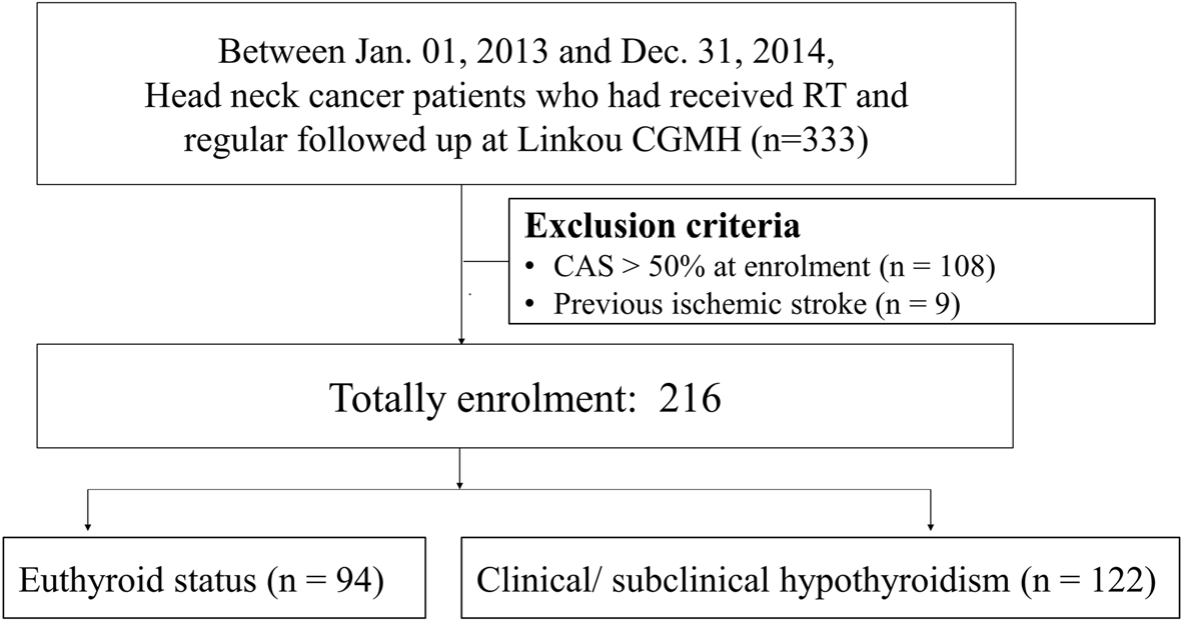
Patient enrolment. CAS, carotid artery stenosis; CGMH, Chang-Gung memorial hospital; RT, radiotherapy

From January 1, 2013 to December 31, 2014, HNC patients who had ever completed RT and received regular follow-up at radio-oncology and neurology departments of Linkou Chang Gung Memorial Hospital were initially prospectively recruited, but the data were retrospectively reviewed. In this study, we aimed to detect the impacts of hypothyroidism in the early phase of post-RT vasculopathy, patients with previous stroke or > 50 % CAS at enrolment were assumed to be vulnerable to vascular events were excluded in this study to improve the homogeneity of cerebrovascular risks of the study population. Demographic data and details of risk factors for common stroke, such as dyslipidemia, hypertension, diabetes mellitus, heart disease, and cigarette smoking, were obtained from all the recruited patients. Laboratory data at enrolment, including glycated hemoglobin and low-density lipoprotein cholesterol, were recorded. Furthermore, medication records, particularly on the use of antiplatelets, statins, or thyroxine, were collected. In addition, all patients received regular thyroid function monitoring at enrolment, including free T4 and thyroid stimulating hormone (TSH) (Fig. 1). We obtained written informed consent from all the participants. The study was approved by the Ethics Institutional Review Board of Chang Gung Memorial Hospital (No. 100-4153B).

### Cancer and RT data

The pathological types and locations of HNC, the extent of lymph node involvement, and cancer staging were recorded. All the patients received photon-beam intensity-modulated radiation therapy (IMRT). IMRT is inherently limited by the physical properties of the photon beam, resulting in unavoidable irradiation of normal tissues at low to moderate doses even at substantial distances from the tumor [15]. In the present study, a minimum distance of 5 mm around the clinical target volume was required to define each planning target volume. Treatment consisted of 70 Grays in 33 fractions over 6 weeks and 3 days; 5 fractions were delivered per week. All targets were treated simultaneously. Total treatment times that were 5 days longer than the scheduled treatment period were considered a major violation [16]. Information on the accumulated total doses of RT and the time interval from the latest RT to study enrolment was ascertained from all the enrolled patients.

### Carotid duplex ultrasound studies

We used Philips HDI 5000 (Wesley Hills, NY, USA) or Acuson Sequoia (Siemens, Munich, Germany) 5–10 MHz real-time B-mode imaging system and a 3.0 MHz pulsed-wave color Doppler spectrum analyzer to follow up the carotid and vertebral arteries after RT. B-mode examinations in the sagittal (anterior–posterior, posterior–anterior, and lateral) and transverse views of the extracranial carotid arteries were used to detect stenotic features. The degree of CAS was defined according to the standard ultrasound criteria [17]. Personnel from our carotid duplex ultrasound (CDU) laboratory diagnosed CAS with an overall accuracy of > 90 % [18]. As total plaque score (TPS) is a known predictor of CAS after RT [19], we also assessed the presence and severity of plaques in each CDU study [19]. In each side, we measured 5 segments, namely the proximal common carotid artery, distal common carotid artery, carotid bifurcation, internal carotid artery, and external carotid artery. In total, we assessed 10 segments bilaterally. The grading scores of the plaques at each site were defined as follows: Grade 0, normal or no plaques; Grade 1, all plaques occupying < 30 % of the vessel diameter; Grade 2, at least 1 plaque occupying 30–49 % of the vessel diameter; Grade 3, at least 1 plaque occupying 50–69 % of the vessel diameter; Grade 4, at least 1 plaque occupying 70–99 % of the vessel diameter; and Grade 5, total occlusion of the vessel. The TPS for each patient was defined as the sum of the plaque scores obtained from the 5 arterial segments in both carotid arteries [19].

### Grouping

Patients were grouped based on their thyroid function at enrolment. Patients with normal TSH (≤ 5 mIU/L) and free T4 level were categorized in the euthyroid (EU) group. Patients with elevated TSH (> 5 mIU/L), including overt HT and subclinical HT, or patients who had already received thyroxine treatment for HT at enrolment were categorized into the HT group.

### Follow‐up strategy and outcomes

All the patients received serial thyroid function (TSH and free-T4) follow-up every 6 months. Furthermore, serial CDU studies were performed annually to monitor CAS progression. The main outcomes of interest in this study were CAS progression and upcoming ischemic stroke (IS). We defined CAS progression as > 50 % stenosis on the B-mode or peak systolic velocities ≥ 120 cm/s based on the hemodynamic criteria at any internal carotid artery or common carotid artery in the follow-up CDU study. The presence of upcoming IS was confirmed by clinical stroke symptoms with compatible brain image findings.

### Statistical analysis

Power analysis was performed to estimate the participant numbers in each group. Alpha error with 0.05, statistical power with 0.9, and allocation ratio with 1 were set up. The estimated proportions of stroke events of the HT and EU groups between 18 and 64 years old were set up with 3.3 % and 2.4 % according to previous study [20]. The estimated sample size was 90 in each group. We used SPSS 22.0 (SPSS, Chicago, IL, USA) to analyze clinical data. We used the Kolmogorov–Smirnov test to examine normality. Parameters were presented as means ± standard deviation or n (%). We used an independent two-sample *t* test to examine differences in continuous data between the study groups. In addition, categorical variables were compared using the chi-square test or Fisher’s exact test. Both event risk and time to event (CAS progression or IS) between the study groups were compared using a multivariable Cox proportional hazards model with adjustments for the selected variables (total doses of RT, time interval from the latest RT, antiplatelet use, age, gender, and smoking) that might confound outcomes. Furthermore, the adjusted curves of time to event for each group were depicted using the multivariable Cox model. Moreover, time to CAS progression and IS were further analyzed during the subgrouping of patients in the HT group based on the use or nonuse of a thyroxine supplement. Statistical significance was set at *p* < 0.05.

## Results

Between January 1, 2013 and December 31, 2014, 333 HNC patients who had ever received RT before and had regular follow-up after RT in our hospital were initially recruited. Nine patients who had IS previously and 108 patients who had > 50 % CAS at enrolment were excluded. Among the 216 patients finally enrolled, 94 (44 %) were categorized into the EU group and the remaining 122 (56 %) who had abnormal thyroid function, including those with clinical and subclinical HT, were categorized into the HT group (Fig. 1). Compared with the EU group, the HT group received high mean total radiation doses (HT vs. EU group: 7021.55 ± 401.67 vs. 6869.69 ± 425.32 cGy, *p* = 0.02) and was less male dominant (HT vs. EU group: 63.9 % vs. 77.7 %, *p* = 0.03). Additionally, the mean age of patients enrolled (HT vs. EU group: 56.15 ± 10.40 vs. 55.41 ± 9.09 years, *p* = 0.56), mean time interval between RT and patient enrolment (HT vs. EU group: 9.18 ± 4.54 vs. 8.65 ± 4.48 years, *p* = 0.39), and baseline TPS at enrolment (HT vs. EU group: 3.30 ± 2.91 vs. 3.43 ± 3.43, *p* = 0.78) were similar between the groups (Table 1).

**Table 1:** Comparison of baseline characteristics between the EU and HT groups

		HT group (*N* = 122)	EU group (*N* = 94)	*p*
Demographics				
Age (y/o)		56.15 ± 10.40	55.41 ± 9.09	0.58
Gender (male, %)		78 (63.9 %)	73 (77.7 %)	0.03*
Hypertension (%)		36 (29.5 %)	37 (39.4 %)	0.13
Diabetes mellitus (%)		20 (16.4 %)	16 (17.0 %)	0.90
Smoking (%)		62 (50.8 %)	59 (62.8 %)	0.08
RT dose (centi-grays)		7021.55 ± 401.67	6869.69 ± 425.32	0.02*
RT interval (years)		9.18 ± 4.54	8.65 ± 4.48	0.39
Baseline total plaque scores		3.30 ± 2.91	3.43 ± 3.43	0.78
Laboratory data				
HbA1C (%)		5.90 ± 0.63	5.83 ± 0.56	0.48
Cr (mg/dL)		0.88 ± 0.27	0.89 ± 0.33	0.80
LDL (mg/dL)		123.11 ± 41.32	119.78 ± 38.93	0.55
Triglyceride (mg/dL)		127.70 ± 88.50	132.64 ± 80.03	0.68
TSH		4.41 ± 3.94	2.33 ± 1.26	< 0.001*
Free-T4		1.06 ± 0.22	1.10 ± 0.16	0.15
High-sensitivity CRP		3.30 ± 6.06	3.62 ± 6.45	0.78
Medications				
Anti-platelets (%)		85 (69.7 %)	55 (58.5 %)	0.09
Statins (%)		19 (15.6 %)	10 (10.6 %)	0.29
Clinical follow-up weeks		190.25 ± 29.00	192.26 ± 33.71	0.64
CDU follow-up weeks		152.39.25 ± 46.38	152.76 ± 42.84	0.95

*CDU* carotid duplex ultrasound, *Cr* creatinine, *EU* euthyroid, *HbA1C* glycated hemoglobin, *HT* hypothyroidism, *LDL* low-density lipoprotein, *RT* radiation therapy, *TSH* thyroid stimulating hormone, *CRP* C-reactive protein; *p < 0.05

The mean follow-up periods were similar between the EU (192.26 ± 33.71 weeks) and HT (190.25 ± 29.00 weeks; *p* = 0.64) groups. Time to CAS progression and time to IS were compared between the study groups. The cumulative incidence plot showed similar trends of CAS progression (adjusted hazard ratio [HR] = 0.57; 95 % confidence interval [CI] = 0.23–1.46; *p* = 0.24) and 

**Fig. 2:**
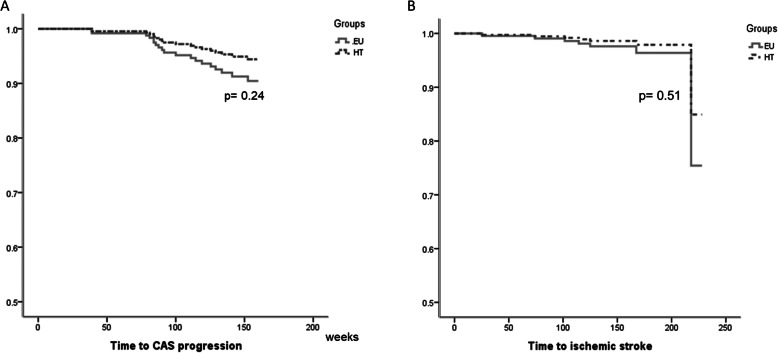
Cumulative time to events between patients with euthyroid (EU) and hypothyroidism (HT). Multivariate-adjusted survival curves comparing the time to (**a**) carotid artery stenosis (CAS) progression and (**b**) ischemic stroke between patients with EU and HT. The incidence rates of CAS progression and ischemic stroke were comparable between the 2 groups

**Fig. 3:**
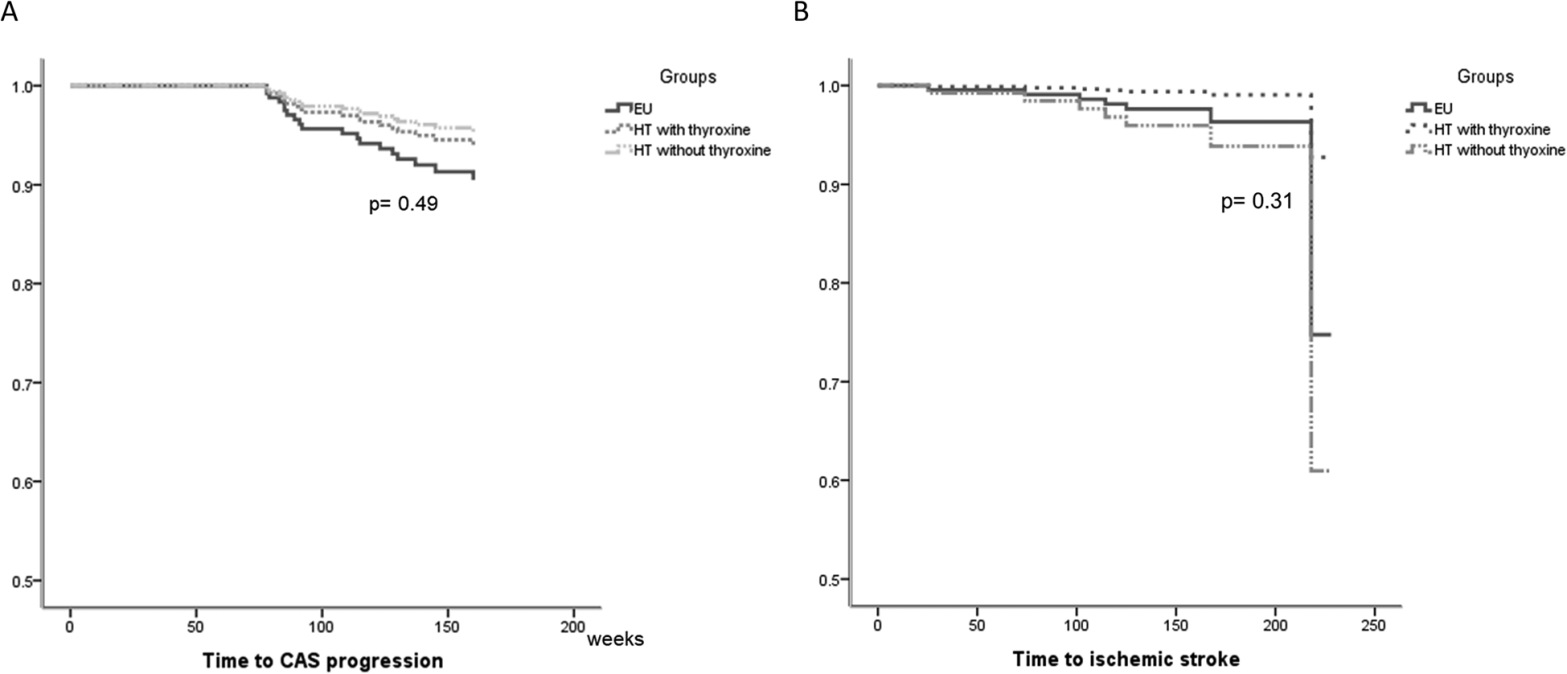
Cumulative time to events between patients with euthyroid (EU), hypothyroidism (HT) with or without thyroxine supplement. Multivariate-adjusted survival curves comparing the time to (**a**) carotid artery stenosis (CAS) progression and (**b**) ischemic stroke between patients in the EU group and HT group with and without thyroxine supplements. The incidence rates of CAS progression and ischemic stroke were similar regardless of thyroxine supplementation

IS (adjusted HR = 0.58; 95 % CI = 0.11–2.94; *p* = 0.51) between the EU and HT groups (Fig. 2). Among the 122 patients of the HT group, 91 (74.6 %) received thyroxine supplement and 31 (25.4 %) did not receive any thyroxine supplement. As thyroxine supplementation in patients with clinical or subclinical HT might influence the clinical outcomes, time to CAS progression and IS were further analyzed when the patients in the HT group were subgrouped based on thyroxine supplement. However, Cox regression analyses did not demonstrate statistical significance in time to CAS progression (*p* = 0.49) or IS (*p* = 0.31) among patients of EU, HT without thyroxine supplement, or HT with thyroxine supplement (Fig. 3). Compared with the EU group, the adjusted HRs of CAS progression and IS were 0.48 (95 % CI = 0.10–2.19; *p* = 0.34) and 1.7 (95 % CI = 0.26–11.03; *p* = 0.58) in the HT group patients with thyroxine supplementation. The adjusted HRs of CAS progression and IS were 0.62 (95 % CI = 0.22–1.72; *p* = 0.36) and 0.26 (95 % CI = 0.03–2.50; *p* = 0.24), respectively, in the HT patients without thyroxine supplement.

## Discussion

Our single-center study did not demonstrate that post-RT HT could further potentiate the risks of CAS progression and IS in patients with post-RT vasculopathy. Similar to previous study, our results also showed patients with post-RT HT received higher radiation doses (Table 1) [3]. Both HT and CAS are common long-term complications of RT in HNC patients. HT may be associated with higher atherogenic and IS risks in young patients. Patients with clinical or subclinical HT are associated with a high frequency of CAS [21]. Subclinical HT or HT could be risk factors for plaque ulceration, and therefore, may lead to increased cerebrovascular events [22]. Moreover, subclinical HT may increase the risk of small vessel disease [23]. Poor thyroid function is also associated with increased risks of fibrosis [24], endothelial dysfunction, and chronic inflammation [25, 26]. It should be necessary to study the association between post-RT HT and post-RT vasculopathy. Our results may add some valuable information to this under-investigated topic.

Post-RT vasculopathy in HNC patients is a common and rapidly progressive complication, but at present, few effective treatments are available to alleviate post-RT vasculopathy in these patients (Table 2) [13, 27, 28]. Every effort to mitigate IS occurrence and the progression of post-RT vasculopathy in HNC survivors is crucial [29]. However, in the early post-RT vasculopathy phase, few interventions had been proved effective in curbing or slowing down disease progression [30–34]. In our results, thyroxine supplement showed insufficient clinical benefits on CAS progression or IS prevention in HNC patients after RT. In patients with post-RT HT, the presence of mild CAS may not be the main indication to start thyroxine treatment. Clinical judgment of thyroxine supplement in overt or subclinical HT is still based on general HT symptoms, such as fatigue, chilliness, depression, cognitive impairment, or body weight gain [35]. Thyroxine supplementation could theoretically alleviate atherosclerosis through the mitigation of cholesterol composition and a decrease in inflammation [5]. However, the effects of thyroxine supplementation on the improvement of intima-media thickness have been controversial in previous reports [8, 36]. In further, thyroxine may attenuate a fibrosis response but may enhance cellular or endothelial proliferation [37–39]. These findings may confound the necessity of HT treatment in post-RT vasculopathy patients. In our study, the effects of thyroxine supplement on the progression of CAS or on the upcoming IS event were not prominent. However, the timing of thyroxine supplement initiation, particularly in subclinical hypothyroidism patients, extent of metabolic syndrome controls, background cerebrovascular status, drug compliance, and dose adjustment during thyroid function fluctuation may all have confounded the study results. Because no good evidence-based treatment against post-RT vasculopathy exists, our results may still add some evidence to this issue. Prospective studies with more patient recruitment and strict vascular risk stratification may be needed in the future to identify the vulnerable subgroups who may gain benefits from thyroxine supplementation.

**Table 2:** Summary of studies investigating the effect of different drug classes on radiation-induced vascular complications

Drug class		Potential mechanism		Animal study results		Real-world study observations
Statin		Inhibits radiation-induced cell death, generates proinflammatory and profibrotic responses in different tissues, and inhibits extracellular matrix deposition in human fibrotic cells [40]		Radiation-induced atherosclerosis could not be circumvented by atorvastatin [31]		Statins were associated with a reduction in the combination of stroke and TIA (HR = 0.4; 95 % CI = 0.2–0.8; *p* = 0.01) [33] Statins were associated with a significant reduction of 32 % in stroke outcome alone (HR = 0.68, 95 % CI = 0.48–0.98, *p* = 0.04) [41]
Antiplatelet		Anti-inflammatory and antithrombotic [30]		Radiation-induced atherosclerosis could not be circumvented by aspirin and clopidogrel [30, 31]		No significant difference in the risk of IS or TIA between patients on continuous oral antithrombotic agents and nonusers (adjusted HR = 0.81; 95 % CI = 0.20–3.31, *p* = 0.77) [34]
Thyroxine		May attenuate fibrosis response [38]		NA		No significant difference in stroke risk among patients with euthyroid or hypothyroidism using or not using thyroxine supplement (*p* = 0.31; our study)
ACE inhibitors		Reduces radiation-induced normal tissue damage [40]		NA		NA
PPAR-γ agonist		Pioglitazone protects the artery wall against irradiation, with decreased plaque surface and less MMP expression [40]		NA		NA
Pentoxifylline + α-tocopherol		Reduces radiation-induced fibrosis [40]		NA		NA

*ACE* angiotensin converting enzyme, *CI* confidence interval, *HR* hazard ratio, *IS* ischemic stroke, *MMP* matrix metalloproteinase, *NA* not available, *PPAR-γ* peroxisome proliferator-activated receptor-gamma, *TIA* transient ischemic attack

Inflammation, fibrosis, and endothelial proliferation play crucial roles in post-RT vasculopathy [12]. Anti-inflammatory, antioxidant, and antifibrotic treatments have been evaluated to reduce the likelihood of this consequence [40]. Table 2 summarizes previous animal and real-world observational studies investigating the effect of different drug classes on radiation-induced vascular complications. Statin seems to be beneficial in some real-world studies [41]. However, the weighting of different mechanisms of statin use, such as a lipid-lowering or anti-inflammatory effect, in ameliorating vascular complication in HNC patients after RT remains uncertain. Therefore, whether statin is beneficial for all patients or only for those with elevated low-density lipoprotein cholesterol is unknown. In our study, the frequency of statin use, levels of low-density lipoprotein and high sensitivity C reactive protein were not statistically different between the HT and EU groups. Our results were insufficient to demonstrate the impacts of lipid-lowering and anti-inflammatory effects of statin on post-RT vasculopathy. Besides, pentoxifylline and α-tocopherol may prevent fibrosis after RT. Although these drugs may improve neck fibrosis after RT, their effect on vascular protection requires further evaluation [42].

Our study had several limitations. First, the number of enrolled patients was too small to demonstrate clinical significance. Real-world studies investigating medication treatments in post-RT vasculopathy usually enrolled more than 1000 patients. However, in claims database studies, HT diagnosis depends on clinician coding. Determining real thyroid function might be difficult, and subclinical HT might not be registered. In future, a prospective cohort study with more patients may help to reduce this limitation and provide clear answers. Second, the TSH and free-T4 level of the same patient may change over time. Patients categorized into the subclinical hypothyroidism initially may develop overt hypothyroidism during follow-up period. Therefore, we combined overt and subclinical hypothyroidism into the same group. This may have possibility to dilute the clinical results. Third, there was no universal protocol for thyroxine management in these recruited patients. There was no general guidance for the initiation of thyroxine supplement in clinical or subclinical HT after RT. The compliance of thyroxine supplement in each patient may confound the study results. Besides, the associated symptoms and vascular risk factors may interfere with the clinician’s decision, and this may also confound the study results. A prospective study with standardized thyroxine treatment protocol can better reveal the real benefits of thyroxine treatments. Third, evaluations of the vascular complication may not be comprehensive, and the follow-up period may not have been sufficiently long in this single-center study. Furthermore, high vascular risk patients with severe CAS were not enrolled. These could have led to an underestimation of the actual clinical events, particularly silent infarctions.

## Conclusions

Interactions between thyroid function, CAS progression, and stroke prevention in HNC patients after RT is complex. So far, no practical clinical guidance exists for this population. Our results did not demonstrate the influence of HT or the clinical benefits of thyroxine supplementation on CAS progression or IS prevention in these patients. Future studies are warranted to assess the vulnerable subgroups whose post-RT HT should be carefully monitored and treated, particularly patients who may have increased post-RT vascular complications.

## Data Availability

The datasets used and/or analyzed during the current study are available from the corresponding author upon reasonable request.

## Abbreviations

CAS: Carotid artery stenosis
CDU: Carotid duplex ultrasound
CI: Confidence interval
EU: Euthyroid
HNC: Head and neck cancer
HR: Hazard ratio
HT: Hypothyroidism
IMRT: Intensity-modulated radiation therapy
IS: Ischemic stroke
RT: Radiotherapy
TPS: total plaque score
TSH: Thyroid stimulating hormone
